# Aortic root and ascending aortic dilatation in patients with repaired tetralogy of Fallot. Determinants, rates of progression, impacts on outcomes and relations to branch pulmonary artery stenosis

**DOI:** 10.1186/1532-429X-15-S1-O101

**Published:** 2013-01-30

**Authors:** Beatrice Bonello, Sonya V Babu-Narayan, Gerhard Diller, Yumi Shiina, Sylvain Beurtheret, Michael Gatzoulis, Daryl Shore

**Affiliations:** 1 Congenital Heart Disease, CHU Timone Marseille, Marseille, France; 2 CMR unit, Royal Brompton Hospital, Londoon, UK; 3 Congenital Heart Disease, Royal Brompton Hospital, London, UK

## Background

We examined features, progression and relations to outcomes of aortic dilatation (AD) in adults with repaired tetralogy of fallot (rtoF).

## Materials and methods

Retrospective study of 110 rtoF adults with native aorta, median age 30.9 (22.9-39.4) years were studied by cardiovascular magnetic resonance (CMR) at baseline, and at follow-up (median 6.3 [IQR:5.1-7.6] years). Aortic measurements were performed at sinus, sino-tubular junction (STJ), and mid-ascending aorta level. AD was defined as diameter > 2 standard deviations larger than published normal Z-Scores, according to the age and body surface area.

## Results

**Figure 1:**
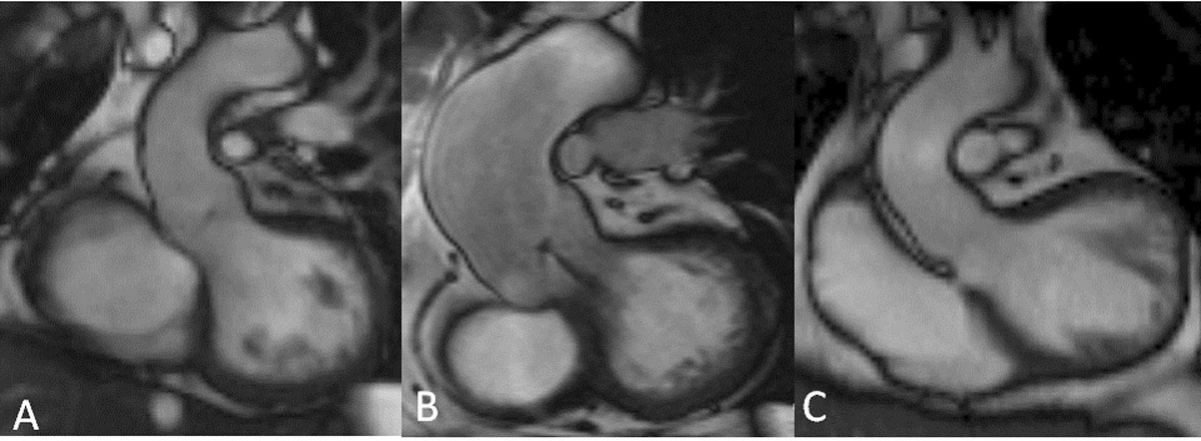


Sixty three percent of patients had AD, sinus dilatation was observed in 61% of the patients (Figure 1A) and 22% of the patients had associated with sinus dilatation, ascending aorta (AscAo) dilatation(Figure 1B). STJ dilatation was present in 44%. Aortic dimensions were normal in 47% of patients (Figure 1C). Predictors of sinus dilatation were age (p=0.009), male gender (p=0.039), previous Blalock-Taussig (BT) shunt (p=0.0004). Independent predictor was previous BT shunt (p=0.004). Predictors of STJ dilatation were pulmonary atresia (PAt) (p=0.02), previous Waterston shunt (p=0.005) and male gender (p=0.009). Independent predictors were PAt (p=0.01) and male gender (p-0.02). Predictors of AscAo dilatation were age at repair (p=0.01), systemic hypertension (p=0,04), PAt (p<0.0001), male gender (p=0.0004). On ROC curve analysis, sinus diameter >39 mm was strongly associated with AscAo (AUC0.90, p<0.0001). Independent predictors of AscAo dilatation were PAt (p<0.0001) and male gender (p=0.004). No patient experienced any aorta related event during follow-up. Diameter increase was observed in 25% of patients at sinus, 21% at STJ and in 35% at AscAo level, at a mean rate of 0.05 ± 0.1, 0.06 ±0.14 and 0.12 ±0.26 mm/year respectively. In those patients, the progression was superior to the one expected according to the normal growth with the age. There was no change of diameter observed in the remainder 53% of patients. No predictors of aortic diameter progression were found.

## Conclusions

Aortic dilatation is common in patients with rtoF but rates of diameter increase in mid-term follow-up are low when assessed by serial CMR. However, when observed, the diameter progression is superior to the one expected with the age. Ascending aorta dilatation is likely when aortic sinus diameter is greater than 39mm. Our data provides guidance on the need of aortic intervention in contemporary adult cohort with rtoF.

## Funding

British Heart Foundation Fellowship (SVB-N).

French Federation of Cardiology (BB).

Unrestricted Actelion educational grant (GD).

The study was supported by the NIHR Cardiovascular Biomedical Research Unit of Royal Brompton and Harefield NHS Foundation Trust and Imperial College London.

